# Commentary: FSH and various forms of FSH receptors: distribution and their functions in gonads and extra-gonadal tissues

**DOI:** 10.1186/s13048-021-00893-y

**Published:** 2021-10-30

**Authors:** Sham S. Kakar, Mariusz Z. Ratajczak

**Affiliations:** 1 Department of Physiology, University of Louisville, Louisville, KY 40202 USA; 2 Present Address: Department of Medicine, University of Louisville, Louisville, KY 40202 USA

FSH is central to reproduction and regulates ovarian production of steroid hormones which in turn regulate endometrial growth/receptivity/regeneration/remodeling. However, these well defined boundaries of the 70s and 80s are now blurring with steroid hormone receptors being reported in the hematopoietic system and FSHR on several extragonadal sites including cancers in multiple tissues [1], and in the endometrium, myometrium, cervix, and placental [2] and hematopoietic system [3, 4]. Female patients being subjected to FSH therapy for stimulating their ovaries show effective mobilization of stem cells in their peripheral blood [5].

Bhartiya’s group reported that FSH treatment enhanced hematopoiesis in the bone marrow by almost 72 h in 5-FU treated mice [6]. How does FSH exerts its pleiotropic effects? What is the significance of FSHR3 and how significant it is functionally compared to the canonical FSHR1? Ovarian cancer cells do not exhibit cAMP signaling upon treatment with FSH [7].

Ratajczak and Bhartiya groups have published data to show FSHR expression on very-small embryonic-like stem cells (VSCLs) [4, 8] in reproductive tissues and surprisingly FSH treatment upregulated alternately spliced FSHR3 more significantly compared to the canonical FSHR1. Similarly, Sullivan et al. [9] reported FSHR3 to be the predominant isoform in sheep ovaries. It did not come as a surprise to see a special issue focused on different facets of FSH/FSHR biology published in 2020 wherein experts raised several concerns regarding extragonadal expression of FSHR [10]. They doubt the results and think these to be due to technical shortfalls [11].

Currently held belief written in golden letters in reproductive biology textbooks is the fact that initial follicle growth is gonadotropin independent and FSHR are not expressed on primordial follicles and that FSH acts on granulosa cells in the ovaries and Sertoli cells in the testes [12].

Bhartiya’s group points this as a ‘misconcept’ and points out this discrepancy has surfaced because of initial studies used only FSHR1 specific primers to detect FSHR in various types of follicles which will not detect FSHR3.

We are pleased to publish two review articles in the Journal of Ovarian Research on FSH/FSHR biology authored by Bhartiya’s group from ICMR-National Institute for Research in Reproductive Health, Mumbai, INDIA. The group has attempted to provide explanation to various concerns raised by Rahman’s group [11]. Since these articles question several basic existing paradigms in the field of reproductive biology including ovarian biology, further discussions and comments will be welcome and we will be pleased to publish in JOVR.

## References

[1] Radu A, Pichon C, Camparo P, Antoine M, Allory Y, Couvelard A (2010). Expression of follicle-stimulating hormone receptor in tumor blood vessels. N Engl J Med.

[2] Stilley JA, Christensen DE, Dahlem KB, Guan R, Santillan DA, England SK (2014). FSH receptor (FSHR) expression in human extragonadal reproductive tissues and the developing placenta, and the impact of its deletion on pregnancy in mice. Biol Reprod.

[3] Ratajczak MZ (2017). Why are hematopoietic stem cells so 'sexy'? On a search for developmental explanation. Leukemia..

[4] Mierzejewska K, Borkowska S, Suszynska E, Suszynska M, Poniewierska-Baran A, Maj M (2015). Hematopoietic stem/progenitor cells express several functional sex hormone receptors-novel evidence for a potential developmental link between hematopoiesis and primordial germ cells. Stem Cells Dev.

[5] Zbucka-Kretowska M, Eljaszewicz A, Lipinska D, Grubczak K, Rusak M, Mrugacz G (2016). Effective mobilization of very small embryonic-like stem cells and hematopoietic stem/progenitor cells but not endothelial progenitor cells by follicle-stimulating hormone therapy. Stem Cells Int.

[6] Shaikh A, Bhartiya D, Kapoor S, Nimkar H (2016). Delineating the effects of 5-fluorouracil and follicle-stimulating hormone on mouse bone marrow stem/progenitor cells. Stem Cell Res Ther.

[7] Li Y, Ganta S, Cheng C, Craig R, Ganta R, Freeman LC (2007). FSH stimulates ovarian cancer cell growth by action on growth factor variant receptor. Mol Cell Endocrinol.

[8] Sriraman K, Bhartiya D, Anand S, Bhutda S (2015). Mouse ovarian very small embryonic-like stem cells resist chemotherapy and retain ability to initiate oocyte-specific differentiation. Reprod Sci.

[9] Sullivan RR, Faris BR, Eborn D, Grieger DM, Cino-Ozuna AG, Rozell TG (2013). Follicular expression of follicle stimulating hormone receptor variants in the ewe. Reprod Biol Endocrinol.

[10] Simoni M, Huhtaniemi I, Santi D, Casarini L (2019). Editorial: follicle-stimulating hormone: fertility and beyond. Front Endocrinol (Lausanne).

[11] Chrusciel M, Ponikwicka-Tyszko D, Wolczynski S, Huhtaniemi I, Rahman NA (2019). Extragonadal FSHR expression and function—is it real?. Front Endocrinol.

[12] Coss D (2020). Commentary on the recent FSH collection: known Knowns and known unknowns. Endocrinology.

